# Supplementary material for: Atomic-scale manipulation of polar domain boundaries in monolayer ferroelectric In2Se3
Source: Nat Commun. 2024 Jan 24;15:718. doi: 10.1038/s41467-023-44642-9 (PMC10808116; doi:10.1038/s41467-023-44642-9)
Supplement: Supplementary file 1 — Supplementary Information [file 41467_2023_44642_MOESM1_ESM.docx]

Supplementary Information for

Atomic-Scale Manipulation of Polar Domain Boundaries in Monolayer Ferroelectric In_2_Se_3_

Fan Zhang^1†‡^, Zhe Wang^2,3#‡^, Lixuan Liu^4,5^, Anmin Nie^5^, Yanxing Li^6^, Yongji Gong^4^, Wenguang Zhu^2,3*^, Chenggang Tao^1⊥*^

^1^Department of Physics, Virginia Tech, Blacksburg, Virginia 24061, United States.

^2^International Center for Quantum Design of Functional Materials (ICQD), Hefei National Research Center for Physical Sciences at the Microscale, University of Science and Technology of China, Hefei 230026, China.

^3^Department of Physics, University of Science and Technology of China, Hefei 230026, China.

^4^School of Materials Science and Engineering, Beihang University, Beijing 100191, China.

^5^Center for High Pressure Science, State Key Laboratory of Metastable Materials Science and Technology, Yanshan University, Qinhuangdao 066004, China.

^6^Department of Physics, University of Texas at Austin, Texas 78712, United States.

^†^Present address: Department of Physics, University of Texas at Austin, Texas 78712, United States.

^#^Present address: Department of Physics, Southern University of Science and Technology, Shenzhen 518055, China.

^⊥^Present address: Center for Nanophase Materials Sciences, Oak Ridge National Laboratory, Oak Ridge, TN 37830, United States.


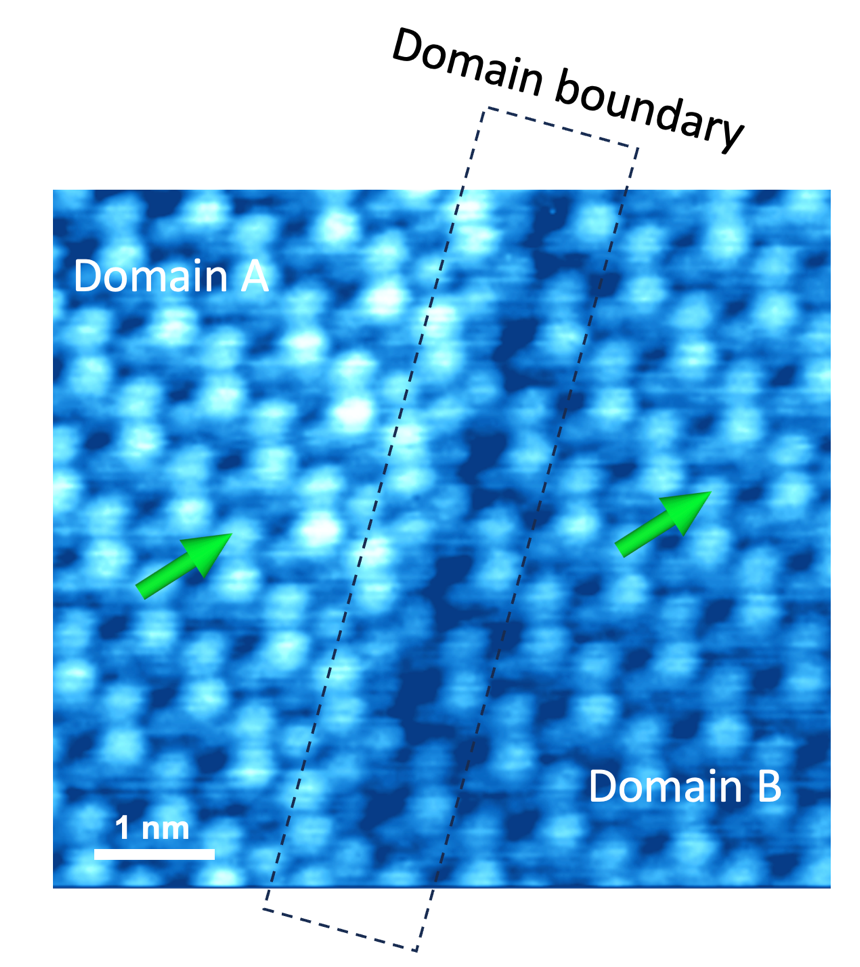


**Supplementary Figure 1. An STM image of a less clearly defined domain boundary (V= 1.5 V, I= 0.4 nA).**


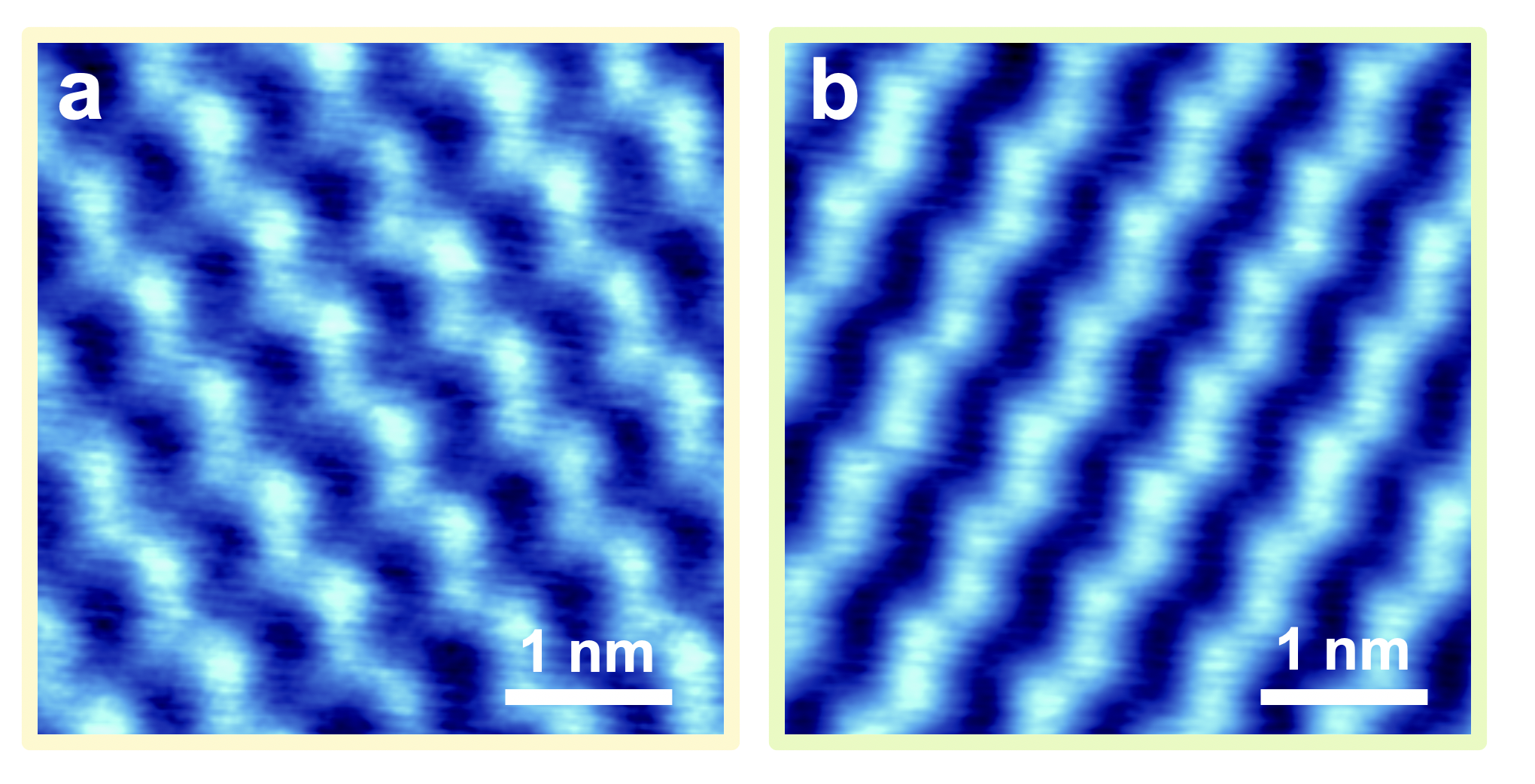


**Supplementary Figure 2. Zoom-in STM images of the areas marked by the green boxes in Figure 1d. (a) Left and (b) Right sides, respectively.**


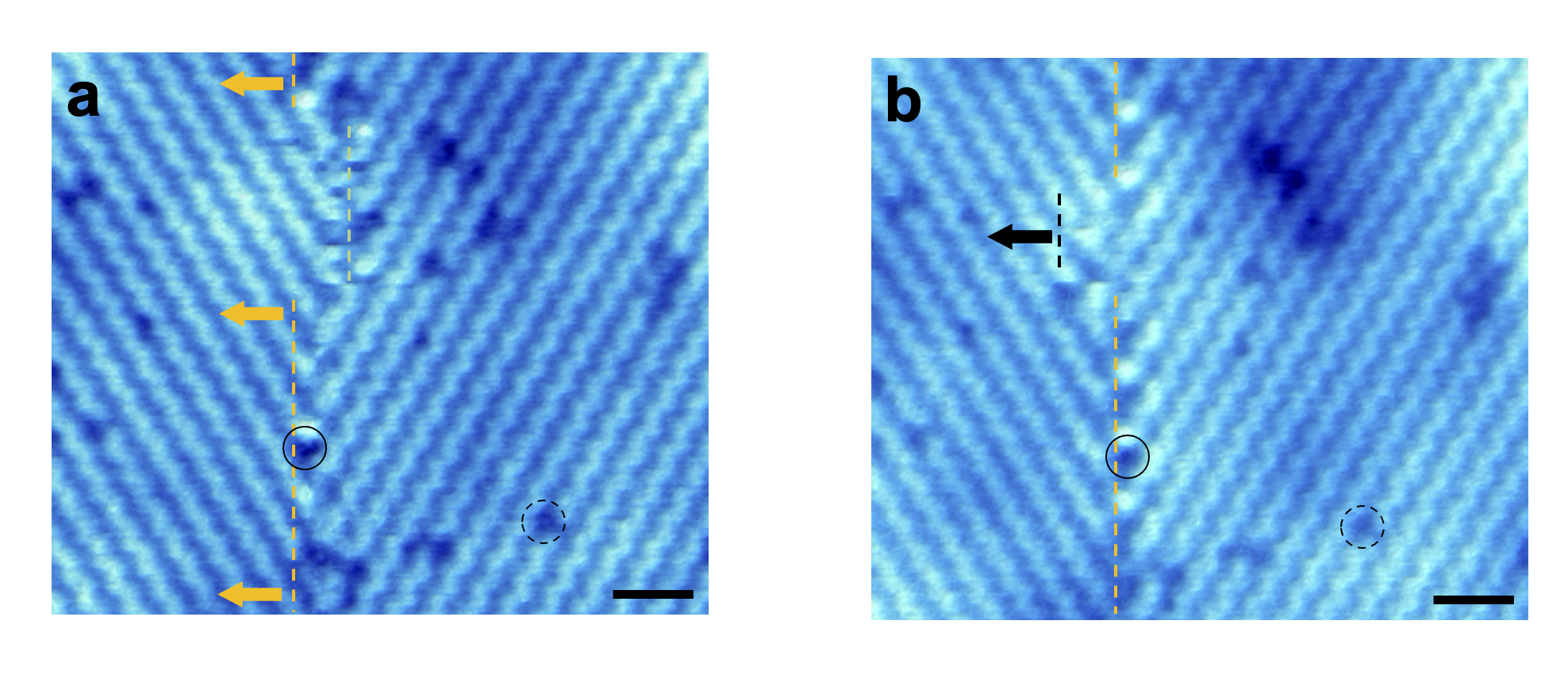


**Supplementary Figure 3. Domain boundary states between two straight configurations during the manipulation process.** STM image of a 60° tail-to-tail domain boundary states (a) between configurations shown in Figures 2a and 2b and (b) in Figures 2b and 2c. Scanning parameters: V_S_ = 4.2 V, I = 0.2 nA, and Scanning speed = 600 s/image. All the scale bars are 2 nm.


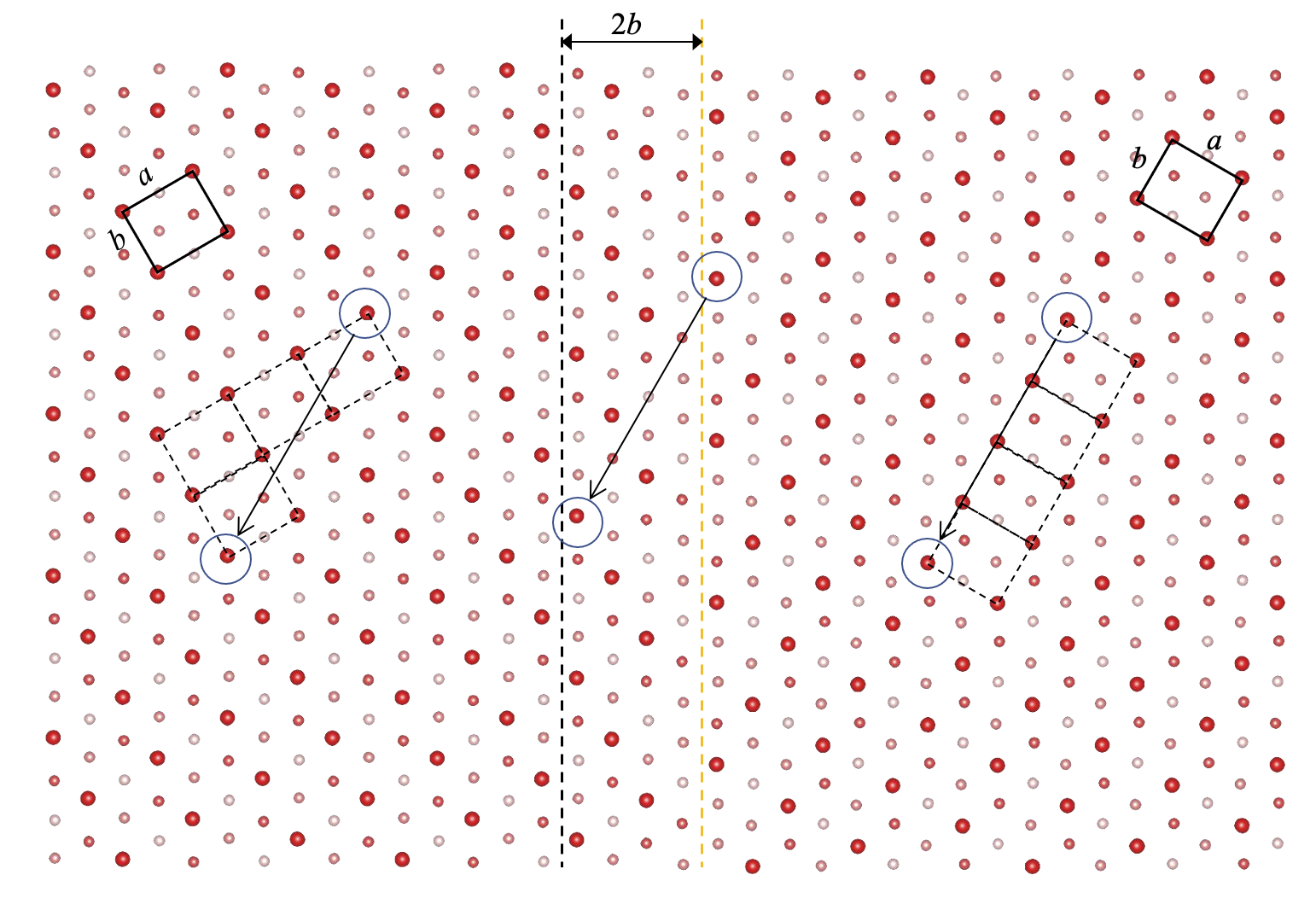


**Supplementary Figure 4. Translational symmetry for the 60°** **tail-to-tail domain boundary structure.** The unit cells of two domains are marked with rectangles and the translation vector is marked with black arrows.


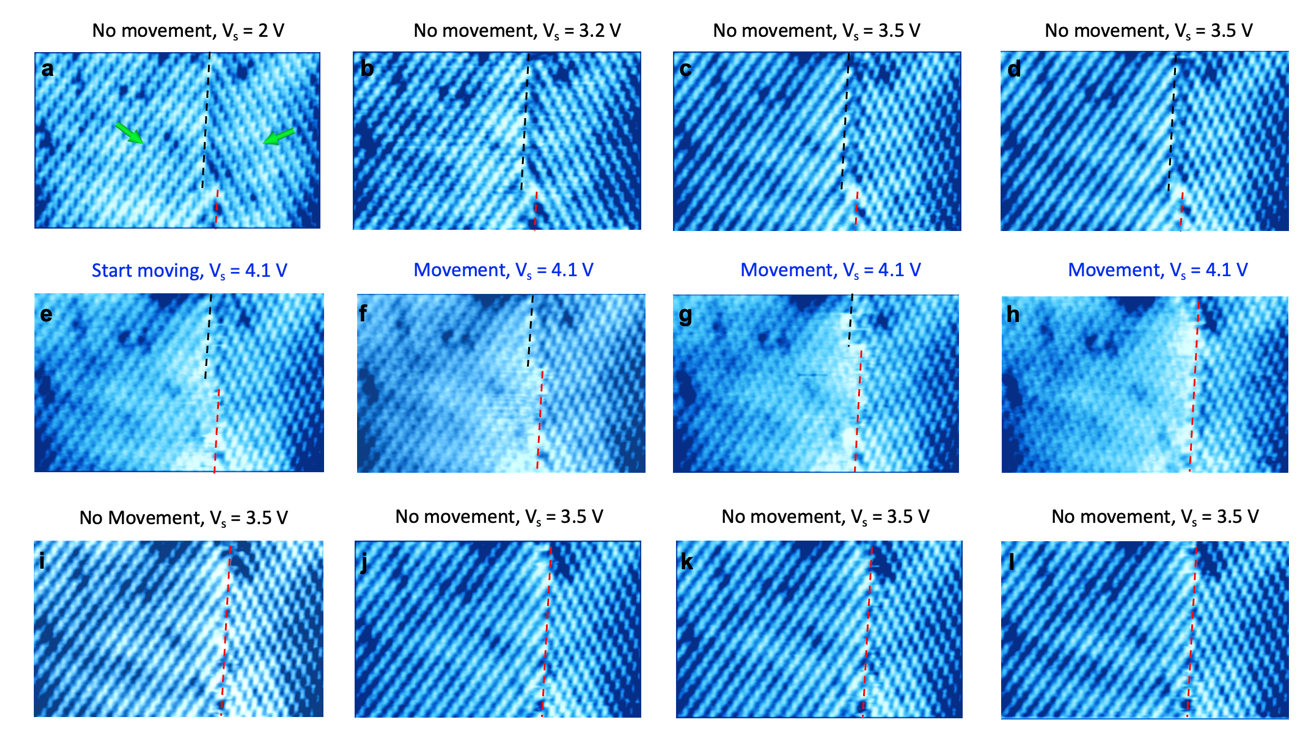


**Supplementary Figure 5. Domain boundary moving dynamics for a head-to-head domain boundary.** (a-l) a sequence of STM images for the same head-to-head domain boundary. The scanning bias for each image is labeled above.

**
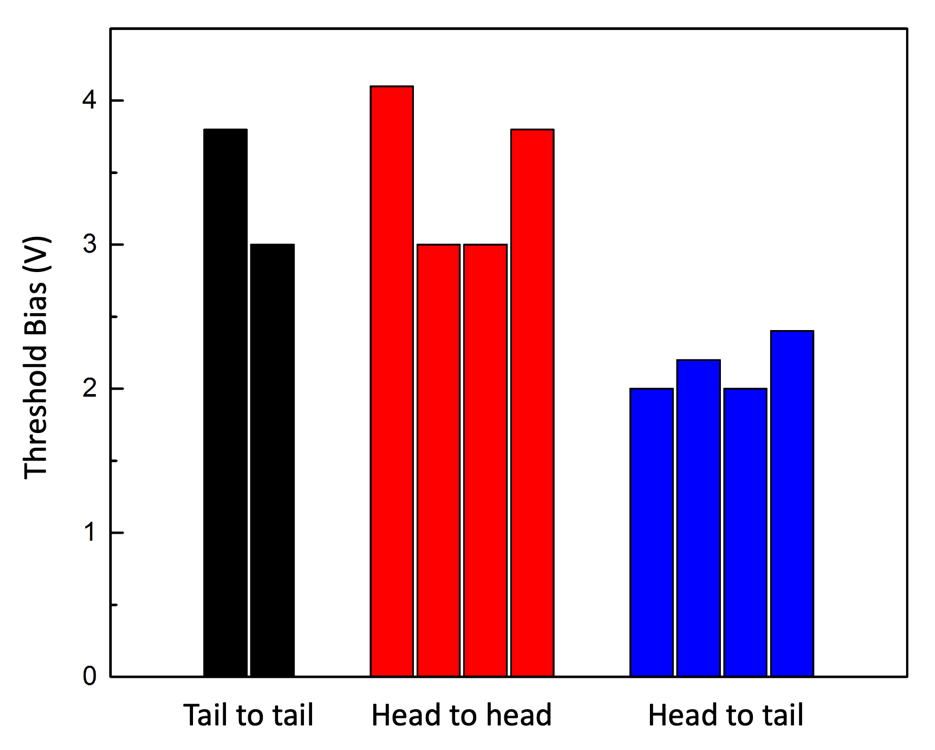
**

**Supplementary Figure 6. The statistic of the threshold bias for manipulating different types of domain boundaries.** The statistic is derived from the manipulation results of two different tail-to-tail domain boundaries, four different head-to-head domain boundaries and four head-to-tail domain boundaries. The variation of the threshold may come from the boundary length and number of defects around the boundary.


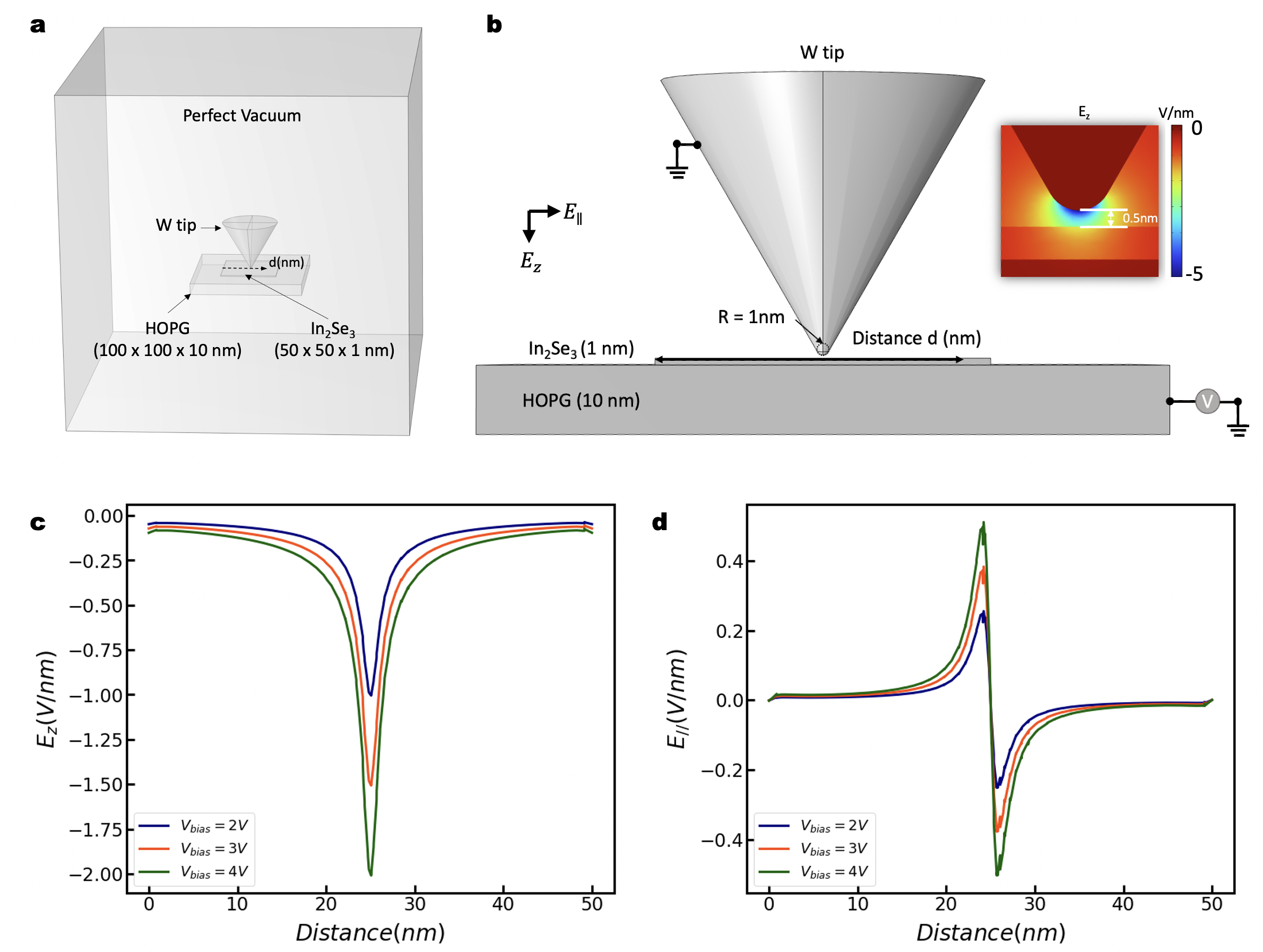


**Supplementary Figure 7. Numerical simulation of tip induced electric field. a-b,** Schematic of the model used for the simulation. The tip was set as grounded, and a voltage of V was applied to the HOPG substrate. The tip is set to be above the center of the In_2_Se_3_ flake and the tip sample distance is set at 0.5 nm. The d axis shown in **a** is parallel to the side and go through the center of the In_2_Se_3_ flake. Other parameters are indicated in the graph. **c-d,** The out-of-plane and in-plane electric fields along a horizontal line along the *d* axis and at the upper surface of In_2_Se_3_ at V_bias_ = 2, 3 and 4 V.


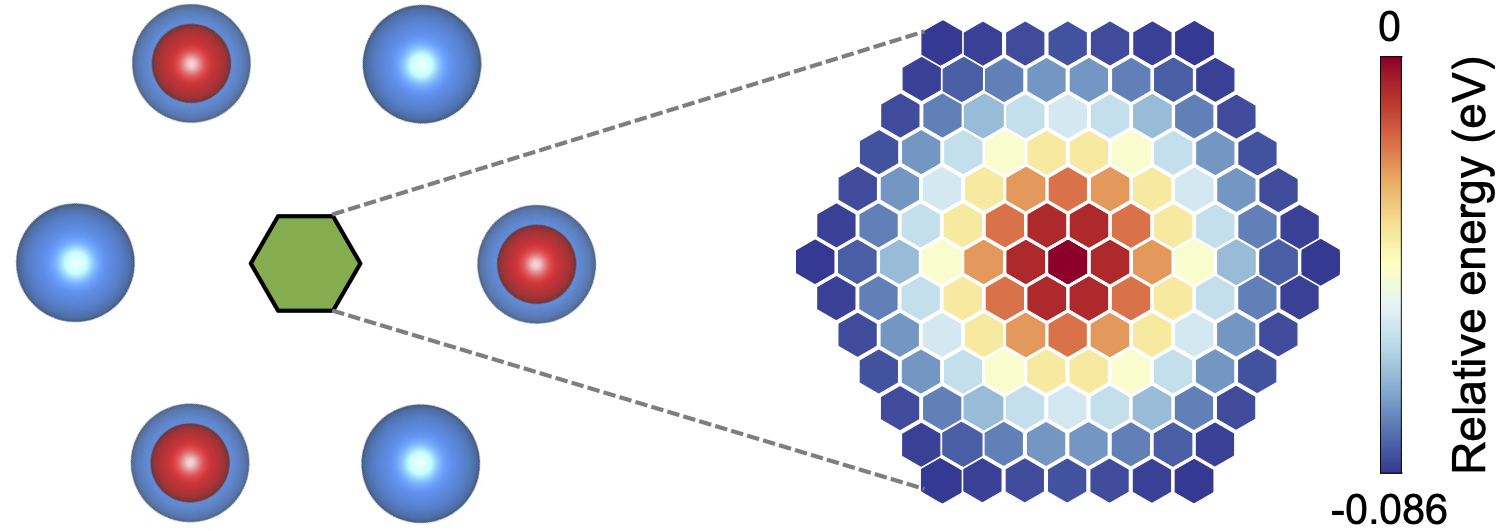


**Supplementary Figure 8. Relative energy map of the central-layer Se atom sampled inside the green hexagon**. The energy map shows the relative energy depending on Se atom’s position in the hexagon. The central-layer Se always tends to deviate from a distance of about 0.5 Å to the hexagon center to maintain a low-energy configuration.


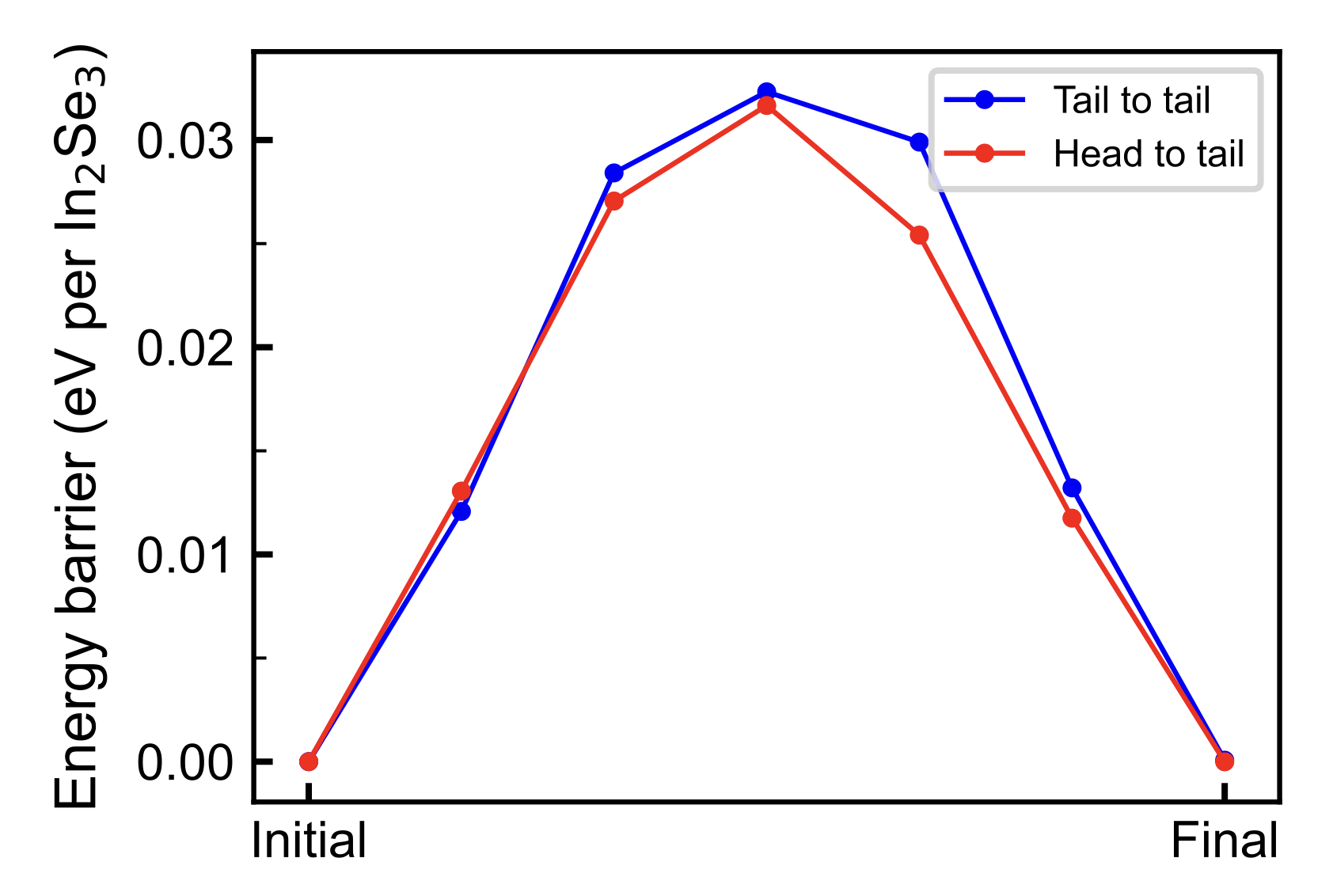


**Supplementary Figure 9. Energy barrier of simulated tail-to-tail and head-to-tail domain boundary shift.**
