# Supplementary material for: Atomic-scale manipulation of polar domain boundaries in monolayer ferroelectric In2Se3
Source: Nat Commun. 2024 Jan 24;15:718. doi: 10.1038/s41467-023-44642-9 (PMC10808116; doi:10.1038/s41467-023-44642-9)
Supplement: Supplementary file 3 — Description of Additional Supplementary Files [file 41467_2023_44642_MOESM3_ESM.pdf]

Description of Additional Supplementary Files

Supplementary Movie 1

The dynamic moving process of a domain boundary during manipulation. It demonstrates that parts of the domain boundary usually move first, and then the whole boundary reaches the new position.

Supplementary Movie 2

The calculated kinetic pathways of a tail-to-tail domain boundary. It shows the optimized structures of the initial, final, and five intermediate states. Each step of the movement involves the collaborative motion of many atoms, and the corresponding energies are shown in Supplementary Figure 9.

Supplementary Movie 3

The calculated kinetic pathways of a head-to-tail domain boundary. It shows the optimized structures of the initial, final, and five intermediate states. Each step of the movement involves the collaborative motion of many atoms, and the corresponding energies are shown in Supplementary Figure 9.
